# Disease of the Fifth Nerve

**Published:** 1881-07

**Authors:** 


### DISEASE OF THE FIFTH NERVE.

A patient fifty years of age had been suffering for fifteen years with a stubborn neuralgia of the second and third branch of the left fifth nerve, as well as of a few fibres of the first branch, the lingual branch being free. Compression of the left carotid alone relieved the attacks, so that the "points douloreux" were no longer sensitive during the period of compression. The patient died of intercurrent disease, and the post mortem showed that the left ganglion gasseri was inclosed in vascular connective tissue, surrounded by a localized pachy-meningitis. Some of the ganglionic cells were pigmented, and the capillaries were much dilated. The patient had received a blow upon the left side of his nose when young, which had set up periostitis, and this inflammation following the trigeminus had localized itself in the middle cranial fossa.

